# Protamine zinc insulin combined with sodium selenite improves glycometabolism in the diabetic KKAy mice

**DOI:** 10.1038/srep26563

**Published:** 2016-05-23

**Authors:** Juan Lu, Wenjun Ji, Mei Zhao, Meng Wang, Wenhui Yan, Mingxia Chen, Shuting Ren, Bingxiang Yuan, Bing Wang, Lina Chen

**Affiliations:** 1 Department of Pharmacology, School of Basic Medical Sciences, Xi’an Jiaotong University Health Science Center, Xi’an 710061, Shaanxi, China; 2 Xi’an No. 1 Hospital, Xi’an 710002, Shaanxi, China; 3 Taizhou People’s Hospital, Taizhou 225300, Jiangsu, China; 4 Department of Pharmacy, 302 Military Hospital of China, Beijing 100039, China; 5 Electron Microscopy Room, Xi’an Jiaotong University Health Science Center, Xi’an 710061, Shaanxi, China; 6 Department of Pathology, School of Basic Medical Sciences, Xi’an Jiaotong University Health Science Center, Xi’an 710061, Shaanxi, China; 7 Key Laboratory of Environment and Genes Related to Diseases (Xi’an Jiaotong University), Ministry of Education, Xi’an 710061, Shaanxi, China

## Abstract

Long-term, high dosage protamine zinc insulin (PZI) treatments produce adverse reactions. The trace element selenium (Se) is a candidate for the prevention of diabetes due to anti-oxidative stress activity and the regulation of glycometabolism. In this study, we aimed to investigate the anti-diabetic effects of a combination of PZI and Se on type 2 diabetes. Diabetic KKAy mice were randomized into the following groups: model group and groups that were subcutaneously injected with PZI, Se, high or low dose PZI + Se for 6 weeks. PZI combined with Se decreased the body weight and fasting blood glucose levels. Moreover, this treatment also improved insulin tolerance, as determined by the reduced values from the oral glucose tolerance test and insulin tolerance test, and increased insulin levels and insulin sensitivity index. PZI combined with Se ameliorated skeletal muscle and β-cell damage and the impaired mitochondrial morphology. Oxidative stress was also reduced. Furthermore, PZI combined with Se upregulated phosphatidylinositol 3-kinase (PI3K) and downregulated protein tyrosine phosphatase 1B (PTP1B). Importantly, the low dosage combination produced effects similar to PZI alone. In conclusion, PZI combined with Se improved glycometabolism and ameliorated the tissue and mitochondrial damage, which might be associated with the PI3K and PTP1B pathways.

Diabetes mellitus is a complex chronic metabolic disease resulting from abnormal insulin secretion, and type 2 diabetes (T2DM) accounts for more than 90% of cases^1^,^2^. T2DM is usually characterized by continuous raised blood glucose levels and multi-organ injury. As the major glucose uptake tissue, skeletal muscle is extremely prone to damage in diabetic mice^3^. A causal relationship between oxidative stress and skeletal muscle damage has been identified under the pathological conditions of diabetes^4^. In eukaryotes, the mitochondrion plays an important role in the respiratory chain, and inevitably produces reactive oxygen species (ROS) as byproducts. Additionally, the mitochondria are also highly dynamic organelles, because changes in their numbers and sizes are closely related to oxidative stress^5^,^6^,^7^. Therefore, medications that reduce oxidative stress or repair mitochondrial damage may be efficacious in treating diabetes^7^.

Insulin is an effective therapy to decrease the blood glucose levels in type 1 diabetes patients and is a selective therapy in type 2 diabetes patients^8^,^9^. A type of man-made insulin, protamine zinc insulin (PZI), has been accepted to enhance the medication safety of insulin^10^. However, exogenetic insulin may cause a strong rejection reaction and drug resistance, which restrains the use of insulin^11^. Thus, drug combinations are needed to increase insulin sensitivity and reduce the insulin dosage. Studies have found that a deficiency in sodium selenium (Se) is positively correlated with the progression of T2DM^12^,^13^. As an essential microelement for human beings, Se has at least two vital roles: an anti-oxidative stress activity and the regulation of glucose transport and glycometabolism^14^. Hence, Se may have potential therapeutic use in treating diabetes. Moreover, it is unknown whether the combination of PZI and Se is efficacious in treating T2DM or whether Se increases the subjects’ sensitivity to PZI.

Pathways mediated by phosphatidylinositol 3-kinases (PI3Ks) are the major signaling pathways involved in the development of diabetes. In addition, dysfunction of protein tyrosine phosphatase (PTP1B) is also strongly related to insulin secretion and signaling^15^,^16^. However, it is not fully understood whether these molecular mechanisms are involved in the effects of PZI and Se.

In this study, yellow KK mice, carrying the yellow obese gene (Ay), were used as a spontaneous diabetic animal model^17^,^18^. Then, the effects of PZI combined with Se in improving T2DM, including myofibril and mitochondria injury, were examined; treatments with the drug combination and high dosage PZI alone were compared. Furthermore, the possible mechanisms underlying the drugs’ applications were also investigated.

## Results

### PZI combined with Se improved the general characteristics and glucose metabolism

**Figure 1:**
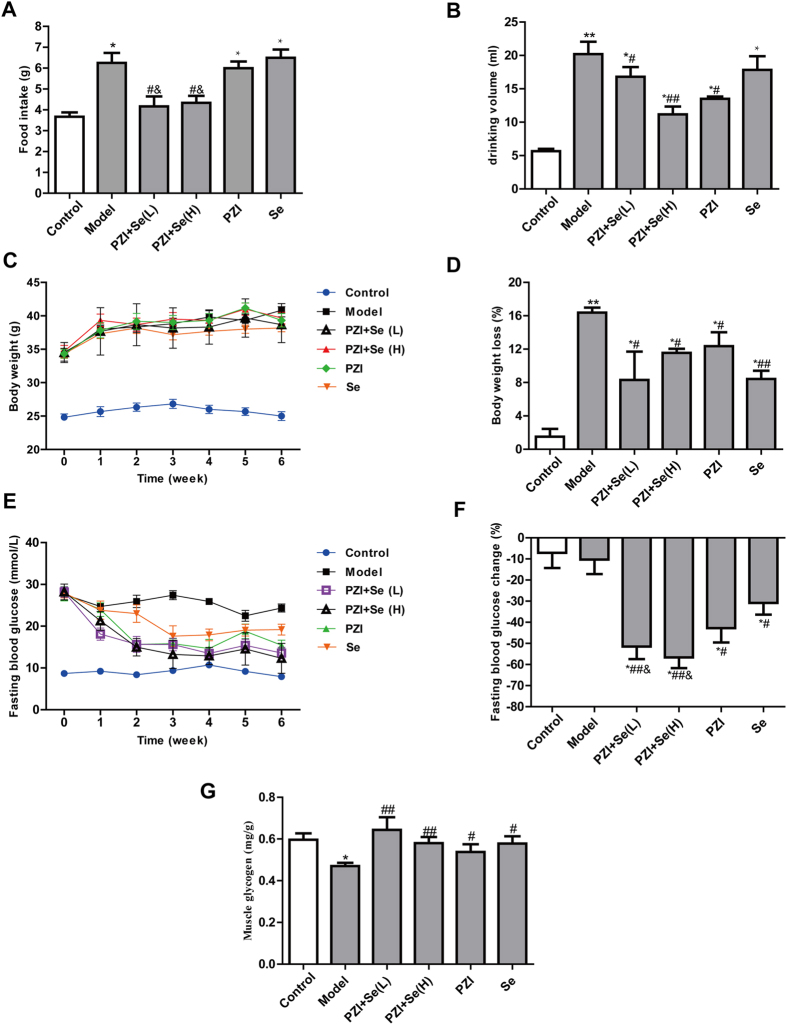
PZI combined with Se improved the general characteristics and glucose metabolism in the KKAy mice. (**A**) Food intake. (**B**) Drinking water volume. (**C**) Body weight (BW). (**D**) Body weight loss. (**E**) Weekly variations in the fasting blood glucose (FBG) levels. (**F**) The decreasing fasting blood glucose levels. (**G**) Muscle glycogen levels. The data are expressed as the mean ± SEM (n = 6). ^*^P < 0.05, ^**^P < 0.01 vs. the control group, ^#^*P* < 0.05, ^##^*P* < 0.01 vs. the model group, ^&^*P* < 0.01 vs. the PZI or Se group. Model: diabetic group; PZI + Se (L): PZI (50 U/kg/d) + Se (100 μg/kg/d); PZI + Se (H): PZI (100 U/kg/d) + Se (200 μg/kg/d).

We have shown that a combination of PZI and Se displayed better performance in STZ-induced diabetic rats (see the supplemental materials). As shown in Fig. 1A,B, a combination treatment consisting of PZI and Se decreased the animals’ food and water intake compared with the diabetic mice. Although an increased BW was observed in the treatment groups compared with the control group, the weight increase in the treatment groups was significantly decreased compared with the model group (Fig. 1C,D).

The baseline fasting blooding glucose (FBG) levels in the KKAy mice were significantly higher than those in the C57 mice, indicating that diabetes had been established. However, PZI, Se, or PZI combined with Se decreased the FBG levels compared with the model group (Fig. 1E). Furthermore, decreases in the FBG levels were rarely observed in the controls and model group, while the levels in the PZI, Se, PZI + Se (H) and PZI + Se (L) groups were 42.79%, 30.85%, 56.63%, and 51.52%, respectively (Fig. 1F). PZI + Se (L) group exhibited a greater regulation of the FBG levels than PZI or Se alone. Glycogen, including hepatic glycogen and muscle glycogen, regulates the glucose level and is an important index. The muscle glycogen level was significantly decreased in the diabetic model group compared with that in the control group. In contrast, the PZI combined with Se group restored the skeletal muscle glycogen level (Fig. 1G).

### PZI combined with Se improved glucose and insulin tolerance

**Figure 2:**
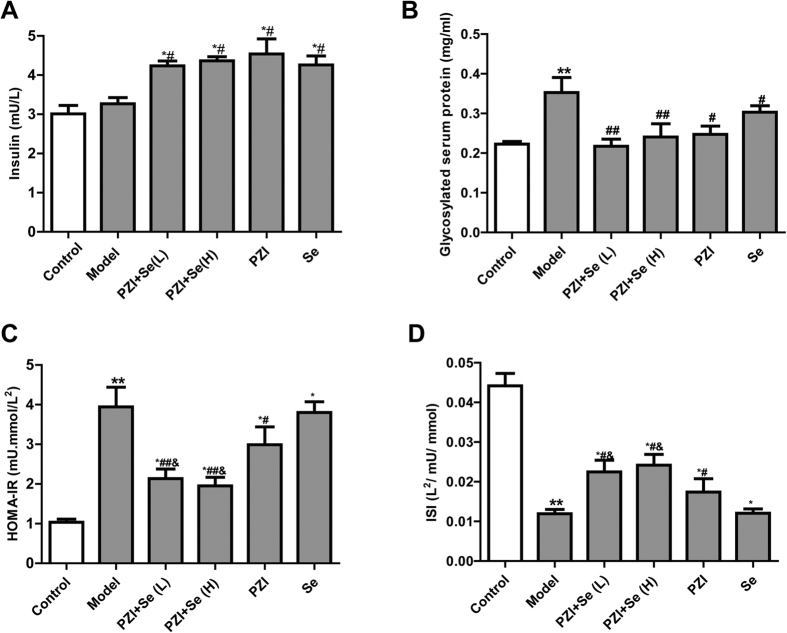
PZI combined with Se improved glucose and insulin tolerance. (**A**) Serum insulin levels. (**B**) Glycosylated serum protein (GSP) levels. (**C**) HOMA-IR. (**D**) ISI. HOMA-IR: homeostasis model assessment of insulin resistance; ISI: insulin sensitivity index. The data are expressed as the mean ± SEM (n = 6). ^**^P < 0.01 vs. the control group, ^#^*P* < 0.05, ^##^*P* < 0.01 vs. the model group, ^&^*P* < 0.01 vs. the PZI or Se group. Model: diabetic group; PZI + Se (L): PZI (50 U/kg/d) + Se (100 μg/kg/d); PZI + Se (H): PZI (100 U/kg/d) + Se (200 μg/kg/d).

Insulin plays an essential role in glucose regulation. In our study, the insulin levels in the PZI combined with Se group were significantly increased compared with the diabetic mice. However, there is no difference between control and model groups, which may implicate the end stage of T2DM in KKAy mice (Fig. 2A). Consistent with the FBG levels, glycosylated serum protein (GSP), a short term biochemical index for FBG, was decreased in the PZI combined with Se groups compared to the model group (Fig. 2B). Moreover, PZI combined with Se decreased the homeostasis model assessment of insulin resistance (HOMA-IR) and increased insulin sensitivity index (ISI) compared with PZI or Se alone (Fig. 2C,D). These results may indicate that a combination of PZI and Se have better effects on general characteristics (such as body weight loss) and glucose metabolism.

**Figure 3:**
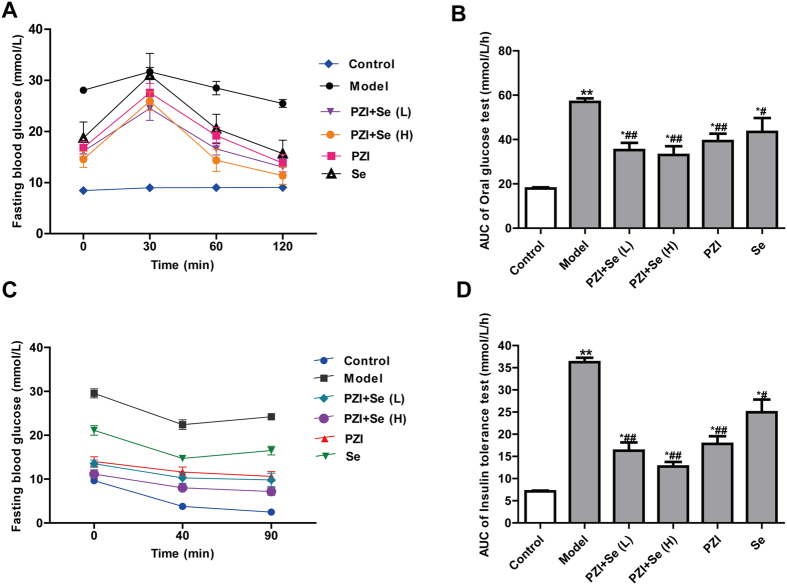
PZI combined with Se improved the results of the tolerance tests. (**A**) FBG levels in the OGTT. (**B**) AUC of the OGTT. (**C**) FBG levels in the ITT. (d) AUC of the ITT. OGTT: oral glucose tolerance test; ITT: insulin tolerance test; AUC: area under the curve. ^**^P < 0.01 vs. the control group, ^#^*P* < 0.05, ^##^*P* < 0.01 vs. the model group. Model: diabetic group; PZI + Se (L): PZI (50 U/kg/d) + Se (100 μg/kg/d); PZI + Se (H): PZI (100 U/kg/d) + Se (200 μg/kg/d).

Oral glucose tolerance test (OGTT) and insulin tolerance test (ITT) are widely used to evaluate glucose and insulin tolerance. In the OGTT, the KKAy mice manifested a higher glucose load at baseline and a strongly elevated load following glucose administration compared with the control group. The glucose load in the treatment groups, including the PZI, Se and PZI combined with Se groups, decreased much more promptly and persisted compared to the model group (Fig. 3A). The AUCs of the OGTT in the treatment groups were significantly smaller than those in the model group (Fig. 3B). In the ITT, the FBG levels in the controls and the treatment groups of PZI, PZI + Se (L), and PZI + Se (H) quickly decreased and stayed the lower levels for 50 min (from 40 min after insulin injection to 90 min at the end of experiment) (Fig. 3C). However, the FBG levels in the model group gradually increased at 90 min after the insulin injection. The AUCs of the ITT in the control and treatment groups were significantly lower than those in the model group (Fig. 3D), indicating that PZI alone, Se alone, or their combination rectified the impaired insulin efficiency. These data revealed that PZI and Se reduced the FBG levels, increased muscle glycogen synthesis, and ameliorated glucose and insulin resistance. Interestingly, the PZI + Se (L) group showed a similar therapeutic effect as the PZI alone group, implying that Se may have increased the animals’ sensitivity to PZI.

### PZI combined with Se improved the ultrastructure of β cells and alleviated the skeletal muscle damage

**Figure 4:**
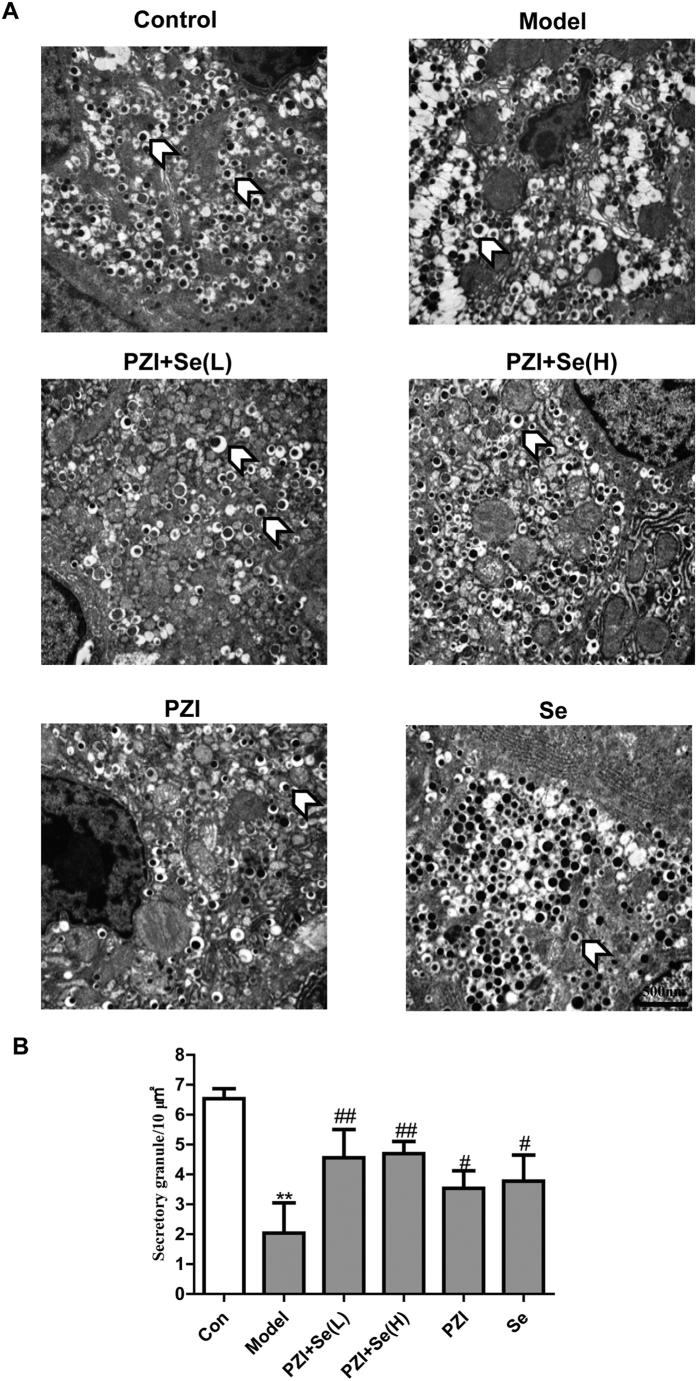
PZI combined with Se improved the ultrastructure of the pancreatic beta cells. (**A**) Representative pictures of pancreatic β cells sections from each group (15,000×). The transparent arrow heads show the secretory granules in the β cell. (**B**) The secretory granules were analyzed in 10 randomly chosen images per group. The data are expressed as the mean ± SEM. ^*^P < 0.05, ^**^P < 0.01 vs. the control group, ^#^*P* < 0.05, ^##^*P* < 0.01 vs. the model group. Model: diabetic group; PZI + Se (L): PZI (50 U/kg/d) + Se (100 μg/kg/d); PZI + Se (H): PZI (100 U/kg/d) + Se (200 μg/kg/d).

An imbalance of energy metabolism, particularly augmented energy intake, leads to an accumulation of lipids in β cells, which triggers glucolipotoxicity. Continuous glucotoxicity and lipotoxicity impair the mitochondria, resulting in inflammation and increased ROS production^4^,^19^. As shown in Fig. 4A, under normal condition, β cells are prevalent in the islets. Normal β cells contain abundant secretory granules with an electron-dense core and clear halo. However, β cells in the diabetic mice revealed less secretory granules, empty vesicles, lipid droplet deposition in the cytoplasm. Swollen mitochondria, destroyed mitochondrial cristae, the development of the Golgi apparatus and an increase in the amount of rough endoplasmic reticulum were also observed in the diabetic group, while these organelles showed few abnormalities in the combination treatment groups. The number of secretory granules per 10 μm^2^ area in the PZI, Se, and PZI combined with Se groups were 3.54, 3.78, 6.70 (H) and 4.56 (L) times the values in the diabetic group. Furthermore, the value of the PZI + Se (H) group was 1.90-fold and that of the PZI + Se (L) group was 1.29-fold higher than the value of the PZI alone group. The value of the PZI + Se (H) group was 1.77-fold and that of the PZI + Se (L) group was 1.21-fold higher than the value of the Se alone group (Fig. 4B).

**Figure 5:**
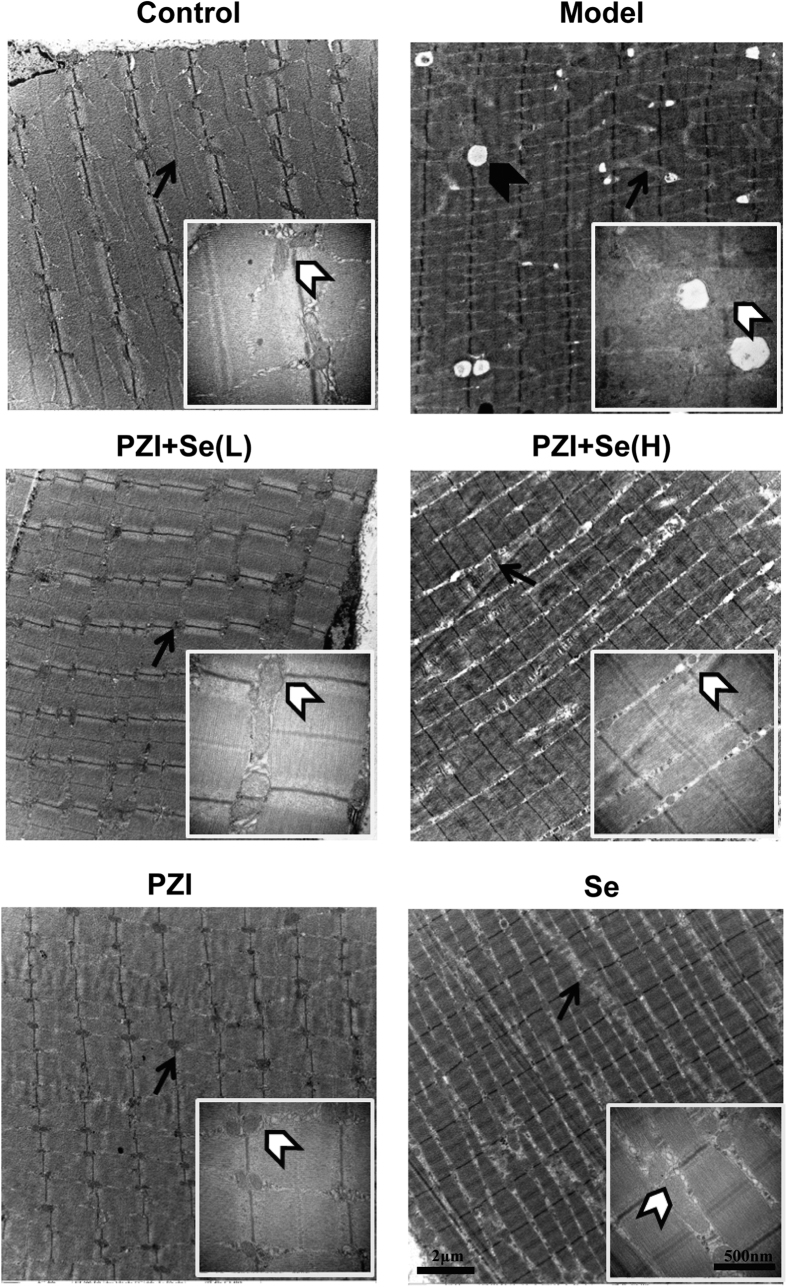
PZI combined with Se improved the ultrastructure of the skeletal muscle tissue. The black arrows show the mitochondria, the transparent arrowheads show the ultrastructure of the mitochondria, and the arrowheads show the lipid droplets. Scale bars: 2 μm and 500 nm. Model: diabetic group; PZI + Se (L): PZI (50 U/kg/d) + Se (100 μg/kg/d); PZI + Se (H): PZI (100 U/kg/d) + Se (200 μg/kg/d).

Skeletal muscle is prone to diabetic injury. The cross sections of the control group were normal, with clear strips and regular myofibrils. An abnormal arrangement and fuzzy strips of myofibrils and vacuoles were observed in the diabetic group. Treatment with PZI and Se alleviated the myofibril atrophy and reduced the numbers of vacuoles (Fig. 5).

### The combination of PZI and Se relieved mitochondria injury and oxidative stress

**Figure 6:**
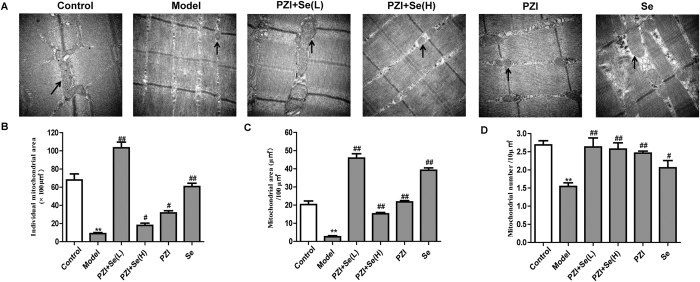
PZI combined with Se improved the mitochondrial dynamics. (**A**) Representative pictures of the mitochondria in each group. The black arrows show the mitochondria in skeletal muscle. Scale bar: 500 nm. (**B**) Area of an individual mitochondrion. (**C**) Mitochondrial area/100 μm^2^. (**D**) Number of mitochondria/10 μm^2^. The data are expressed as the mean ± SEM (n = 6). ^**^P < 0.01 vs. the control group, ^#^*P* < 0.05, ^##^*P* < 0.01 vs. the model group. Model: diabetic group; PZI + Se (L): PZI (50 U/kg/d) + Se (100 μg/kg/d); PZI + Se (H): PZI (100 U/kg/d) + Se (200 μg/kg/d).

To determine whether the mitochondria are involved in diabetes, the morphology of the mitochondria was first observed. As shown in Fig. 6A, disordered mitochondria with fuzzy cristae and lipid droplets were distributed in the skeletal muscle of the model group. In contrast, the lipid droplets were reduced and mitochondrial injury was significantly improved in the treatment groups. The mitochondrion is a highly dynamic organelle that undergoes biogenesis, fissure and fusion following various stresses, including oxidative stress. Compared with the model group, the area of an individual mitochondrion in the skeletal muscle of the control group was increased 7.51-fold, while it was increased 3.31-fold in the PZI group, 6.49-fold in the Se group, 2.35-fold in the PZI + Se (H) group, and 11.4-fold in the PZI + Se (L) group. Of these, the area of an individual mitochondrion was the largest in the PZI + Se (L) group (Fig. 6B). The mitochondrial area/100 μm^2^ in the skeletal muscle was markedly increased in the PZI group (+8.50-fold), Se group (+15.75-fold), PZI + Se (H) group (+4.25-fold) and PZI + Se (L) group (+18.73-fold) compared with the model group (Fig. 6C). The number of mitochondria/10 μm^2^ was higher in the control group (+1.75-fold), PZI group (+1.60-fold), Se group (+3.33-fold), PZI + Se (H) group (+1.67-fold) and PZI + Se (L) group (+1.71-fold) than that in the model group (Fig. 6D).

**Figure 7:**
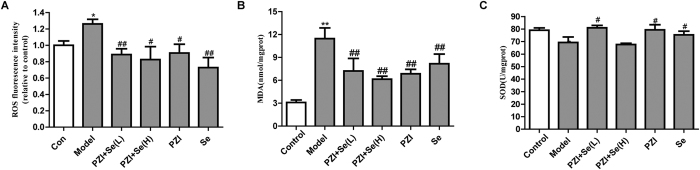
Effects of PZI and Se on oxidative stress. (**A**) ROS levels. (**B**) MDA levels. (**C**) SOD levels. The data are expressed as the mean ± SEM (n = 6). ^**^P < 0.01 vs. the control group, ^#^*P* < 0.05, ^##^*P* < 0.01 vs. the model group. Model: diabetic group; PZI + Se (L): PZI (50 U/kg/d) + Se (100 μg/kg/d); PZI + Se (H): PZI (100 U/kg/d) + Se (200 μg/kg/d).

Oxidative stress plays a critical role in the development of diabetes. Antioxidants (SOD)/oxidants (MDA and ROS) have been used to detect oxidative stress^4^,^6^,^13^. As shown in Fig. 7A, ROS level in diabetic group was higher than control, while treatment groups inhibited ROS. The MDA level in the model group was increased compared with the control group; however, SOD activity was decreased, suggesting that oxidative stress may participate in diabetes. In addition, oxidative stress could be reduced in the combination of PZI and Se group (Fig. 7).

### Effects of PZI combined with Se on regulating the PI3K and PTP1B signaling pathways

**Figure 8:**
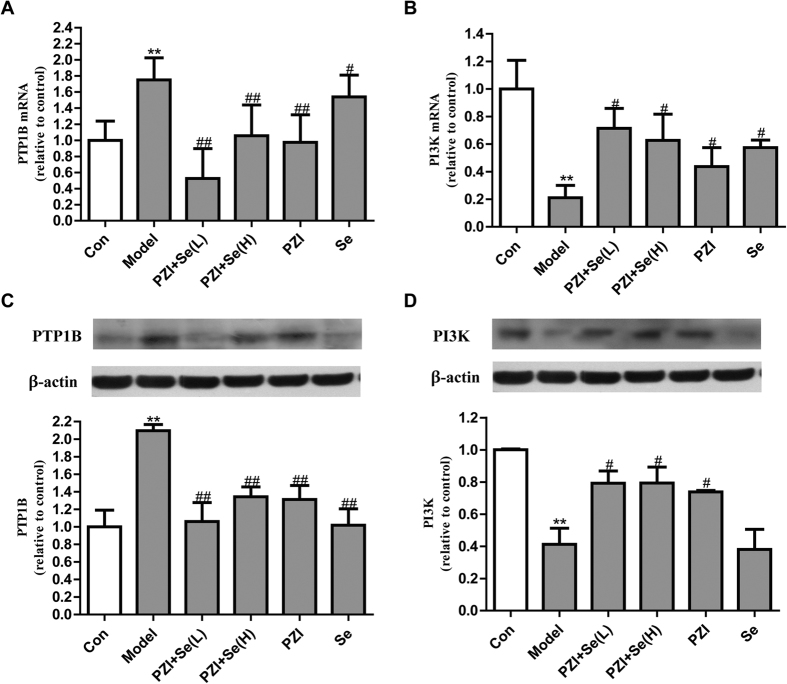
PZI combined with Se influenced the expression levels of the PTP1B and PI3K mRNAs and proteins in skeletal muscle. (**A**) PTP1B mRNA level. (**B**) PI3K mRNA level. (**C**) PTP1B protein level. (**D**) PI3K protein level. The data are expressed as the mean ± SEM (n = 6). ^**^P < 0.01 vs. the control group, ^#^*P* < 0.05, ^##^*P* < 0.01 vs. the model group. Model: diabetic group; PZI + Se (L): PZI (50 U/kg/d) + Se (100 μg/kg/d); PZI + Se (H): PZI (100 U/kg/d) + Se (200 μg/kg/d).

Our results showed that the PTP1B mRNA was upregulated in response to diabetes, while the PI3K levels were decreased in the model group (Fig. 8A,B). In parallel with the mRNA levels, the protein expression levels were also changed accordingly (Fig. 8C,D). Following treatment with PZI, Se, and their combination, the PTP1B levels were decreased and the PI3K levels were increased. The combination of PZI and Se had similar regulatory effects as either monotherapy.

## Discussion

Despite the abundant research regarding the use of insulin in diabetes, the long-term application of insulin is associated with devastating side effects. As a result, physicians prefer to use adjunct methods that are suitable for long-term use. Our study first confirmed that a low dosage of PZI (a half dosage of PZI alone) combined with Se significantly improved the animals’ general characteristics (food and water volume and decrease in BW), decreased the FBG levels, alleviated glucose and insulin resistance, and mitigated skeletal muscle and mitochondrial injury, accompanied by an increase in the PI3K levels and downregulation of PTP1B. Therefore, we highlight a new, safer therapeutics concept that a low dosage of insulin combined with the trace element selenium can be used for long-term diabetes treatment.

Clinical reports have testified that long-term insulin usage in diabetes patients will lead to adverse reactions^20^. Hence, it is important to control the dosage of insulin in diabetes patients. Selenium is an ingredient of various proteins, such as glutathione peroxidase. The protective roles of Se, such as upregulation of Bcl-2, cancer prevention and activation of cytokines, are well accepted. Recently, Se has been found to promote glucose transport and metabolism through an insulin-like effect^21^,^22^. Therefore, we chose a weighted modification method to design the two-drug regimen with PZI and Se. It is a type of multi-factor and multi-level data analysis approach using the U6 (6^6^) uniform design and 6 × 6 optimal Latin square principles that precisely determines the proportion ratio and largely decreases the number of experimental groups^23^. Uniform design is created by Chinese mathematicians Fang KT, to make the test factors entirely and evenly distributed in the experimental arrangement, and makes proportion ratio precise and largely decreases animal numbers^24^. We found that PZI and Se have additive hypoglycemic effects against diabetes. PZI was the main drug, Se was adjuvant, and the theoretical optimization formulation is 1.00:1.75 ≈ 1.0:2.0. Therefore, we chose to treat the diabetic KKAy mice with PZI:Se = 1.0:2.0 for convenience.

PZI combined with Se decreased the FBG levels and restored the muscle glycogen levels, implying that PZI and Se improved glucose metabolism. The net BW in the treatment group was not decreased compared with the diabetic group, but weight loss was attenuated. Weight loss is associated with hyperglycemia; therefore, attenuated weight loss is due to the presence of normal glucose levels. Sheng *et al.*^25^ found that a 1 week sodium selenite (2 mg/kg/d) treatment in Kun-Ming mice with insulin-dependent diabetes mellitus decreased their BW. The difference might be associated with the type of animal, the curative dosage and the duration of treatment. Consistent with the FBG levels, increases in the muscle glycogen and insulin levels and a decrease in the GSP were observed in the treatment groups compared with the diabetic mice. To further indicate the beneficial effects of PZI and Se, glucose homeostasis and insulin sensitivity were both detected. Elevated insulin sensitivity contributes to the repair of abnormal insulin action. Notably, the low dosage of PZI combined with Se showed similar effects as PZI alone, suggesting that Se fulfills the role of the reduced insulin dosage.

β cells dysfunction is the main risk factor correlated with T2DM^7^. T2DM patients treated with neutral protamine Hagedorn’s globin insulin (0.23 U/kg/d)^26^ or T1DM patients treated with isophanuminsulinum (0.1 U/kg/d)^27^ both efficiently improved β cells activity and alleviated hypoglycemia. Schatz *et al.* found that a high dosage of isophanuminsulinum promoted β cells recess^28^ and relieved T1DM. Thus, it seems that PZI combined with Se possibly relieved β cells injury, resulting in increased insulin levels. The KKAy mice showed enriched lipogenesis and inflammatory cell infiltration, while the drug regimen normalized the β cells’ morphology and ameliorated the skeletal muscle damage. In addition, the number of secretory granules per 10 μm^2^ area was increased in the treatment groups, further supporting the recovery of β cells function.

Emerging evidence has indicated that multiple pathophysiological processes are activated by hyperglycemia and ultimately lead to oxidative stress^4^,^13^. Oxidative stress is even present in T2DM. Excessive ROS production plays a vital role in the initiation of skeletal muscle damage in diabetes^29^. The *in vivo* skeletal muscle oxidative capacity is determined by the intrinsic mitochondrial function and the number of mitochondria. Therefore, diabetes causes skeletal muscle injury^30^. In our study, we observed severe mitochondrial damage in the model group, while the groups treated with a combination of PZI and Se exhibited further improvement than those treated with either drug alone. Furthermore, there was a higher level of SOD in the control and medicated groups, and the MDA and ROS levels were lower than the model group. Based on these results, we speculate that the combination of PZI and Se might alleviate mitochondrial and skeletal muscle damage and protect against oxidative stress.

Both PZI and Se have been found to activate the insulin receptor/PI3K/AKT signaling cascade, as well as the subsequent activation of GLUT4 and inhibition of ROS production^19^,^31^,^32^. In addition, PTP1B has been reported to negatively modulate insulin signaling by dephosphorylating insulin and the insulin receptor^33^. It is interesting that PZI and Se share a common signaling pathway in skeletal muscle that includes the activation of PI3K and inhibition of PTP1B.

In conclusion, this is the first study to investigate the protective effects of a combination of PZI and Se on T2DM. Our results indicated that PZI combined with Se at least partially contributed to β cell repair and reduced insulin resistance, skeletal muscle damage and oxidative stress, which may be associated with the upregulation of PI3K and the downregulation of PTP1B. Importantly, a low dosage of PZI combined with Se produced similar effects as PZI alone, and Se might serve as an insulin sensitizer. Therefore, a combination of PZI and Se may be an effective alternative therapeutic treatment for T2DM, when the mechanisms are sufficiently investigated.

## Methods

### Animals

Spontaneous diabetic KK/Upj-Ay/J (KKAy) mice (also called yellow KK mice), an ideal animal model for diabetes research, were developed by crossing the Ay/a mutation onto the inbred KK strain of native Japanese mice^17^,^18^,^34^. They are characterized by obesity, hyperinsulinemia, hyperglycemia, hypertriglyceridemia, hypercholesterolemia and insulin resistance^17^,^18^,^35^,^36^. The frequency of hyperglycemic tends to increase with age^17^. Therefore, the 11–13-week-old male KKAy mice (obtained from Chinese Academy of Medical Science, Beijing, China) and male C57BL/6J mice age-matched with KKAy mice were chosen in the experiment. They were housed individually under a 12 h light-dark cycle under controlled humidity (50 ± 5%) and temperature (20–25 °C), with free access to water and food. A high-fat chow containing 6% fat, 8% water, 18% protein, and 5% fiber (Huafukang Bioscience Company, Beijing, China) was provided to the KKAy mice, and the C57 BL/6J mice were provided with an ordinary rodent diet.

### Experimental protocols

All of the animal experimental procedures were approved by the Ethics Committees of Xi’an Jiaotong University and were reviewed and performed in accordance with the Guideline of Animal Care and Use Committee of Xi’an Jiaotong University. For choosing an appropriate dosage of PZI, we first tried the dosages of 0.5, 1, 4, 10, 50 U/kg/d PZI alone for 3 days, however, they had no significant effect on FBG. The decreasing rates of FBG were lower than 10%. The decreasing rates of FBG of 100 U/kg/d and 200 U/kg/d PZI were about 15% and 30%. In consideration of long-term medication and the aim of the experiment, we chose 100 U/kg/d PZI alone as the positive control. Six male C57BL/6J mice served as the Control group. After a 1 week acclimation, the KKAy mice with fasting blood glucose (FBG) levels > 16.7 mmol/L were randomly assigned into 5 groups (n = 8 per group): (1) Model group, in which the KKAy mice were treated with an equivalent volume of saline; (2) PZI group, in which the KKAy mice were subcutaneously (s.c.) injected with PZI (100 U/kg/d) (purchased from Jiangsu Wanbang Biochemistry Medicine Company, Xuzhou, China); (3) Se group, which was s.c. injected with Se (200 μg/kg/d) (purchased from Shanghai Tiancifu Bioengineering Company, Shanghai, China); (4) PZI + Se (high dose, H) group, which was s.c. injected with PZI (100 U/kg/d) and Se (200 μg/kg/d); and (5) PZI + Se (low dose, L) group, which was s.c. injected with PZI (50 U/kg/d) and Se (100 μg/kg/d). The ratio of PZI and Se was 1:2, which was determined through a uniform design using SD rats (see the supplemental materials). The control group was also treated with an equivalent volume of saline. Six weeks later, the mice were euthanized. The skeletal muscle tissue was harvested for western blot, electron microscopy and RNA extraction.

### Oral glucose tolerance test (OGTT)

At the end of treatment and after a 6 h fast, a 2 g/kg glucose solution was orally administered, and then the blood glucose levels in the blood from the tail veins were measured at 0, 30, 60 and 120 min using a glucose meter. The AUC was calculated for the FBG levels observed during the OGTT.

### Insulin tolerance test (ITT)

After a 6 h fast, the mice were subcutaneously injected with 4 U/kg insulin (Jiangsu Wanbang Biochemistry Medicine Company, Xuzhou, China), and the blood glucose levels were measured at 0, 40 and 90 min after injection using a glucose meter. The AUC was calculated for the FBG levels observed during the ITT.

### Biochemical analysis

The serum insulin levels were measured with an enzyme-linked immune sorbent assay (ELISA) using a mouse insulin ELISA kit (R&D Systems, Inc., Minneapolis, MN, USA). The homeostasis model assessment of insulin resistance (HOMA-IR) and insulin sensitivity index (ISI) were calculated according to the following formulas: HOMA-IR = FBG (mmol/L) × FINS (IU/ml)/22.5 and ISI = 1/[FBG (mmol/L) × FINS (IU/ml)].

Separated sera were used for the estimation of GSP using GSP assay kit (colorimetric method; Serial No: A037). Samples of 100 mg skeletal muscle were homogenized in 2 mL of a cold 0.1 M phosphate buffer with a pH value of 7.4. Tissue homogenates were prepared in a glass tissue homogenizer. The homogenate was centrifuged at 12000 rpm for 15 min and the supernatant was used for the measurement of glycogen, ROS, SOD and MDA using liver/muscle glycogen assay kit (colorimetric method; Serial No: A043), ROS assay kit (chemiluminescence; Serial No: E004), total SOD assay kit (hydroxylamine method; Serial No: A001) and MDA assay kit (thibabituric acid method; Serial No: A003). GSP, glycogen, SOD and MDA were determined with an ELISA reader (Thermo Fisher Scientific, Inc., Waltham, MA, US) and ROS were measured with fluorescence microplate reader (PerkinElmer, Inc., Waltham, MA, US) according to the manufacturer’s instructions (Nanjing Jiancheng Bioengineering Institute, Nanjing, China).

### Morphology and ultrastructural examination

The splenic regions of the pancreas were minced into 1 mm cubes, fixed with 2.5% glutaraldehyde and 2% osmic acid, then dehydrated and embedded in epoxy resin. Ultrathin sections were collected onto 200-mesh copper grids, double stained with uranyl acetate and lead acetate, then observed with a Hitachi H-7650 transmission electron microscope (Hitachi, Tokyo, Japan).

The skeletal muscle tissue was prefixed in phosphate buffer (pH 7.2) and embedded in paraffin. Semi-thin sections (1 mm × 1 mm) were examined using a transmission electron microscope (TE2000, Nikon) at 50,000× magnification, as previously reported^37^. The number and size of the mitochondria were analyzed with ImageJ 1.42q software. Eight images were randomly chosen from each group.

### Real-time PCR

The total RNA was isolated from the skeletal muscle using TRIzol reagent. The reverse transcription was performed with 4 μg of RNA using Prime Script™ RT Master Mix (Takara Bio, Inc., Tokyo, Japan). Gene expression was analyzed by real-time quantitative PCR. The primers used in this experiment are listed in Table 1. GAPDH served as an endogenous control and was used to normalize the levels of the tested genes.

### Western blot

One hundred milligrams of thigh tissue was homogenized in 1 mL of RIPA lysis buffer and proteinase inhibitors. Sixty micrograms of the proteins were separated on a 10% SDS/PAGE gel and transferred to PVDF membranes. The membranes were then blocked with 5% non-fat milk or 5% BSA and incubated with primary antibodies against PI3K (p85α) (5405-1, CST, USA), PTP1B (2066-1, Epitomics, UK), and β-actin (sc-47778, Santa Cruz Biotechnology, CA) overnight. After an incubation with the secondary antibody, the bands were treated with horseradish peroxidase (HRP) (Thermo, USA) and then visualized using ECL-Plus reagent (Pierce, Thermo Corporation, USA). The bands were analyzed with ImageJ 1.42q.

### Statistical analysis

The data are shown as the means ± SEM. Normal distribution analysis before ANOVA analysis was made with SPSS 16.0 for windows (SPSS Inc, Chicago). Skewness < 1 and Kurtosis < 1 were considered as normal distribution. Differences between groups were analyzed by ANOVA followed by Tukey’s multiple comparison test using GraphPad Prism Version5.01 (GraphPad Software Inc., La Jolla, CA, USA). *P* values less than 0.05 were considered statistically significant.

## Additional Information

**How to cite this article**: Lu, J. *et al.* Protamine zinc insulin combined with sodium selenite improves glycometabolism in the diabetic KKAy mice. *Sci. Rep.* **6**, 26563; doi: 10.1038/srep26563 (2016).

## Supplementary Material

Supplementary Information

## Figures and Tables

**Table 1:** Primer sequences used for real-time PCR.

Gene	Forward primer	Reverse primer
PI3K	5′-GCTCCTGGAAGCCATTGAGAA-3′	5′-CGTCGATCATCTCCAAGTCCAC-3′
PTP1B	5′-CACAGTACGACAGTTGGAGTTGGAA-3′	5′-CAGGCCATGTGGTGTAGTGGA-3′
GAPDH	5′-TGTGTCCGTCGTGGATCTGA-3′	5′-TTGCTGTTGAAGTCGCAGGAG-3′

## References

[b1] Zhang B. B. & Moller D. E. New approaches in the treatment of type 2 diabetes. Curr Opin Chem Biol 4, 461–467 (2000). 10959776 10.1016/s1367-5931(00)00103-4

[b2] Taylor R. Type 2 diabetes: etiology and reversibility. Diabetes Care 36, 1047–1055 (2013). 23520370 10.2337/dc12-1805 PMC3609491

[b3] Zierath J. R., Houseknecht K. L., Gnudi L. & Kahn B. B. High-fat feeding impairs insulin-stimulated GLUT4 recruitment via an early insulin-signaling defect. Diabetes 46, 215–223 (1997). 9000697 10.2337/diab.46.2.215

[b4] Kowluru R. A. & Mishra M. Oxidative stress, mitochondrial damage and diabetic retinopathy. Biochim Biophys Acta 1852, 2474–2483 (2015). 26248057 10.1016/j.bbadis.2015.08.001

[b5] Holloway G. P., Gurd B. J., Snook L. A., Lally J. & Bonen A. Compensatory increases in nuclear PGC1alpha protein are primarily associated with subsarcolemmal mitochondrial adaptations in ZDF rats. Diabetes 59, 819–828 (2010). 20103701 10.2337/db09-1519 PMC2844829

[b6] Valero T. Mitochondrial biogenesis: pharmacological approaches. Curr Pharm Des 20, 5507–5509 (2014). 24606795 10.2174/138161282035140911142118

[b7] Chen L. N. *et al.* Liraglutide ameliorates glycometabolism and insulin resistance through the upregulation of GLUT4 in diabetic KKAy mice. Int J Mol Med 32, 892–900 (2013). 23877319 10.3892/ijmm.2013.1453

[b8] Yaturu S. Insulin therapies: Current and future trends at dawn. World J Diabetes 4, 1–7 (2013). 23493823 10.4239/wjd.v4.i1.1 PMC3596776

[b9] Turner R. C., Cull C. A., Frighi V. & Holman R. R. Glycemic control with diet, sulfonylurea, metformin, or insulin in patients with type 2 diabetes mellitus: progressive requirement for multiple therapies (UKPDS 49). UK Prospective Diabetes Study (UKPDS) Group. JAMA 281, 2005–2012 (1999). 10359389 10.1001/jama.281.21.2005

[b10] Herrmann B. L. *et al.* Comparison of insulin aspart vs. regular human insulin with or without insulin detemir concerning adipozytokines and metabolic effects in patients with type 2 diabetes mellitus. Exp Clin Endocrinol Diabetes 121, 210–213 (2013). 23512415 10.1055/s-0033-1334905

[b11] Arnetz L., Rajamand E. N., Hoybye C., Brismar K. & Alvarsson M. Improved Insulin Sensitivity during Pioglitazone Treatment Is Associated with Changes in IGF-I and Cortisol Secretion in Type 2 Diabetes and Impaired Glucose Tolerance. ISRN Endocrinol 2013, 148497 (2013). 23401789 10.1155/2013/148497 PMC3562586

[b12] Yan X. *et al.* Dietary selenium deficiency partially rescues type 2 diabetes-like phenotypes of glutathione peroxidase-1-overexpressing male mice. J Nutr 142, 1975–1982 (2012). 23014491 10.3945/jn.112.164764 PMC3497934

[b13] Zhang C. *et al.* Diabetes-induced hepatic pathogenic damage, inflammation, oxidative stress, and insulin resistance was exacerbated in zinc deficient mouse model. Plos One 7, e49257 (2012). 23251339 10.1371/journal.pone.0049257 PMC3520990

[b14] Higuchi A., Inoue H., Kawakita T., Ogishima T. & Tsubota K. Selenium compound protects corneal epithelium against oxidative stress. Plos One 7, e45612 (2012). 23049824 10.1371/journal.pone.0045612 PMC3458096

[b15] Lungkaphin A. *et al.* Impaired insulin signaling affects renal organic anion transporter 3 (Oat3) function in streptozotocin-induced diabetic rats. Plos One 9, e96236 (2014). 24801871 10.1371/journal.pone.0096236 PMC4011703

[b16] Stull A. J. *et al.* Skeletal muscle protein tyrosine phosphatase 1B regulates insulin sensitivity in African Americans. Diabetes 61, 1415–1422 (2012). 22474028 10.2337/db11-0744 PMC3357297

[b17] Nakamura M. & Yamada K. Studies on a diabetic (KK) strain of the mouse. Diabetologia 3, 212–221 (1967). 4907141 10.1007/BF01222198

[b18] Iwatsuka H., Shino A. & Suzuoki Z. General survey of diabetic features of yellow KK mice. Endocrinol Jpn 17, 23–35 (1970). 5468422 10.1507/endocrj1954.17.23

[b19] Mason S. & Wadley G. D. Skeletal muscle reactive oxygen species: a target of good cop/bad cop for exercise and disease. Redox Rep 19, 97–106 (2014). 24620937 10.1179/1351000213Y.0000000077 PMC6837413

[b20] Wangnoo S. K., Ghosal S., Akhtar S., Shetty R. & Tripathi S. Clinical experience of switching from glargine or neutral protamine Hagedorn insulin to insulin detemir in type 2 diabetes: Observations from the Indian cohort in the A1chieve study. Indian J Endocrinol Metab 18, 715–720 (2014). 25285292 10.4103/2230-8210.139239 PMC4171898

[b21] Stranges S. *et al.* A prospective study of dietary selenium intake and risk of type 2 diabetes. Bmc Public Health 10, 564 (2010). 20858268 10.1186/1471-2458-10-564 PMC2949772

[b22] Erbayraktar Z., Yilmaz O., Artmann A. T., Cehreli R. & Coker C. Effects of selenium supplementation on antioxidant defense and glucose homeostasis in experimental diabetes mellitus. Biol Trace Elem Res 118, 217–226 (2007). 17916924 10.1007/s12011-007-0037-5

[b23] Zheng Q. S. & Sun R. Y. Quantitative design of drug compatibility by weighted modification method. Zhongguo Yao Li Xue Bao 20, 1043–1051 (1999). 11270973

[b24] Fang K. T. The uniform design: Application of number-theoretic methods in experimental design. Acta Math Appl Sinica 3, 363–372 (1980). (Chinese).

[b25] Sheng X. Q., Huang K. X. & Xu H. B. Influence of alloxan-induced diabetes and selenite treatment on blood glucose and glutathione levels in mice. J Trace Elem Med Biol 18, 261–267 (2005). 15966575 10.1016/j.jtemb.2005.01.001

[b26] Niskanen L. *et al.* Comparison of a soluble co-formulation of insulin degludec/insulin aspart vs biphasic insulin aspart 30 in type 2 diabetes: a randomised trial. Eur J Endocrinol 167, 287–294 (2012). 22660026 10.1530/EJE-12-0293 PMC3400040

[b27] Hassan K., Rodriguez L. M., Johnson S. E., Tadlock S. & Heptulla R. A. A randomized, controlled trial comparing twice-a-day insulin glargine mixed with rapid-acting insulin analogs versus standard neutral protamine Hagedorn (NPH) therapy in newly diagnosed type 1 diabetes. Pediatrics 121, e466–e472 (2008). 18299307 10.1542/peds.2007-1679

[b28] Schatz D. *et al.* Preservation of C-peptide secretion in subjects at high risk of developing type 1 diabetes mellitus–a new surrogate measure of non-progression? Pediatr Diabetes 5, 72–79 (2004). 15189492 10.1111/j.1399-543X.2004.00047.x

[b29] Stull A. J. *et al.* Skeletal muscle protein tyrosine phosphatase 1B regulates insulin sensitivity in African Americans. Diabetes 61, 1415–1422 (2012). 22474028 10.2337/db11-0744 PMC3357297

[b30] Sartorius T. *et al.* Cinnamon extract improves insulin sensitivity in the brain and lowers liver fat in mouse models of obesity. Plos One 9, e92358 (2014). 24643026 10.1371/journal.pone.0092358 PMC3958529

[b31] Wessels B., Ciapaite J., van den Broek N. M., Nicolay K. & Prompers J. J. Metformin impairs mitochondrial function in skeletal muscle of both lean and diabetic rats in a dose-dependent manner. Plos One 9, e100525 (2014). 24950069 10.1371/journal.pone.0100525 PMC4065055

[b32] Myers S. A., Nield A. & Myers M. Zinc transporters, mechanisms of action and therapeutic utility: implications for type 2 diabetes mellitus. J Nutr Metab 2012, 173712 (2012). 23304467 10.1155/2012/173712 PMC3530793

[b33] Salmeen A. & Barford D. Functions and mechanisms of redox regulation of cysteine-based phosphatases. Antioxid Redox Signal 7, 560–577 (2005). 15890001 10.1089/ars.2005.7.560

[b34] Castle C. K., Colca J. R. & Melchior G. W. Lipoprotein profile characterization of the KKA(y) mouse, a rodent model of type II diabetes, before and after treatment with the insulin-sensitizing agent pioglitazone. Arterioscler Thromb 13, 302–309 (1993). 8427865 10.1161/01.atv.13.2.302

[b35] Ohshima K. *et al.* Direct angiotensin II type 2 receptor stimulation ameliorates insulin resistance in type 2 diabetes mice with PPARgamma activation. Plos One 7, e48387 (2012). 23155382 10.1371/journal.pone.0048387 PMC3498306

[b36] Li Y. *et al.* The DPP-4 inhibitor MK0626 and exercise protect islet function in early pre-diabetic kkay mice. Peptides 49, 91–99 (2013). 24025600 10.1016/j.peptides.2013.08.021

[b37] Ji W. *et al.* Liraglutide Exerts Antidiabetic Effect via PTP1B and PI3K/Akt2 Signaling Pathway in Skeletal Muscle of KKAy Mice. Int J Endocrinol 2014, 312452 (2014). 25183970 10.1155/2014/312452 PMC4144308

