# Supplementary material for: Protamine zinc insulin combined with sodium selenite improves glycometabolism in the diabetic KKAy mice
Source: Sci Rep. 2016 May 23;6:26563. doi: 10.1038/srep26563 (PMC4876423; doi:10.1038/srep26563)
Supplement: Supplementary Information [file srep26563-s1.doc]

**Title page**

**Title:** Protamine zinc insulin combined with sodium selenite improves glycometabolism in the diabetic KKAy mice

**Short title:** A combination of PZI and Se improves glycometabolism

**Authors:** Juan Lu ^a,b,1^, Wenjun Ji ^a,c,1^, Mei Zhao ^a,d,1^, Meng Wang ^a^, Wenhui Yan ^a^, Mingxia Chen ^e^, Shuting Ren ^f^, Bingxiang Yuan ^a^, Bing Wang ^e^ and Lina Chen ^a, g *^

**Affiliations:**

^a^ Department of Pharmacology, School of Basic Medical Sciences, Xi’an Jiaotong University Health Science Center, Xi’an 710061, Shaanxi, China

^b^ Xi’an No. 1 Hospital, Xi’an 710002, Shaanxi, China

^c^ Taizhou People’s Hospital, Taizhou 225300, Jiangsu, China

^d^ Department of Pharmacy, 302 Military Hospital of China, Beijing 100039, China

^e^ Electron Microscopy Room, Xi’an Jiaotong University Health Science Center, Xi’an 710061, Shaanxi, China

^f^ Department of Pathology, School of Basic Medical Sciences, Xi’an Jiaotong University Health Science Center, Xi’an 710061, Shaanxi, China

^g^ Key Laboratory of Environment and Genes Related to Diseases (Xi’an Jiaotong University), Ministry of Education, Xi’an 710061, Shaanxi, China

^1^ These authors contributed equally to this manuscript

*** Corresponding author:**

Lina Chen

Tel.: +86-182 2055 4189

Fax.: +86-29-82655032

E-mail address: chenlin@mail.xjtu.edu.cn (L. Chen)

**Supplemental materials and methods**

**Animals.** 78 male SD rats, 160-180g (provided by the Experimental Animal Center of Xi’an Jiaotong University, China) were used in accordance with the recommended guidelines on the care and use of laboratory animals issued by the Chinese Council on Animal Research. The study was approved by the ethics committee of Xi’an Jiaotong University. All rats were housed in a temperature-controlled room (19℃) and raised under a 12h light/dark cycle with free access to food and water.

**Experimental protocols with uniform design.** After one week’s adaptation, all rats were randomly divided intocontrol (n = 8) and diabetic model (n = 70). Rats in control group were fed with a standard laboratory diet (carbohydrates: 30%, proteins: 22%, lipids: 12%, vitamins: 3%) *ad libitum*, while diabetic rats with high-fat and high-glucose diet (10% refined lard, 20% sucrose, 2.5% cholesterol, 1% sodium cholate and 66.5% common food) provided by Laboratory Animal Center of Xi’an Jiaotong University for 6 weeks. Then diabetic rats were fasting overnight and given a peritoneal injection of freshly prepared streptozotocin (STZ, 25mg/kg body weight; Sigma, St Louis, USA) in 0.1 M citrate buffer (pH 4.5), while the control group was given an equivalent volume of citric acid buffer. 4 weeks after STZ injection, 56 rats with fasting blood glucose (FBG) >16.7 mmol/L measured by a glucose meter (Roche Diagnostics GmbH, Mannheim, Germany) indicated diabetes.

56 diabetic rats were randomly divided into seven groups (n=8 in each group): diabetesmodel and diabetes treated with PZI and Se by hypodermic injection (G1-6 groups) for another 4 weeks. Drugs dosage was designed by Uniform Design's Table of U6 (6^6^) (supplementary Table 1). The equivalent volume of saline was administered to control and model groups. FBG was measured weekly.

**Supplemental results**

**1. PZI combined with Seimprovedgeneral characteristics and FBG in diabetes**

Control group was in good performance, shown as lively movements, robust bodies, luster fur, and soft, thick, clean hair. However, diabetic rats were weak, sweaty, dirty with lackluster fur and thin hair, sluggish to stimuli, more drinking and urine volume etc. FBG levels were stable between 22-30 mmol/L in diabetic rats. PZI and Se treatment significantly improved general characteristics of diabetic rats in the above aspects.FBG levels were reduced following different dosages of PZI andSe.As shown in supplementary Table 1, the decreasing ranges of FBG changed from (35.8±16.5) % in G1 (group 1) to (87.9±1.6) % in G5 (group 5), suggesting that combination of PZI and Se were effective in diabetes treatment.

**2. Optimizationformulations analysis**

The optimal proportion of compound preparation was analyzed by DAS1.0 software. d represents standardized dose and b as the coefficient of standardized dose, the value of b(d) reflects the relative importance of each component. The greater the value of b(d) is, the more significant the dose-dependent manner is. The component with maximum b(d) acts as the main drug. b(d1d2) means the interaction between the two drugs, the greater the value is, the better the synergistic effect is. As shown in supplementary Table 2, PZI served as the main drug, Se was adjuvant, and the theoretical optimization formulation was 1.00:1.75 (PZI 4 U/kg/d: Se 7μg/kg/d). The ratio approximately equals to 1.0: 2.0, therefore we chose 1.0: 2.0 as the ratio of PZI: Se for the next study in KKAy mice.

**Supplementary Table 1.** Effects of PZI and Se combination on FBG in diabetic rats

| Group | PZI (U/kg/d) | Se  (μg/kg/d) | FBG (mmol/L) | | | | | Decreasing rate (%) |
| --- | --- | --- | --- | --- | --- | --- | --- | --- |
|  |  |  | Before treatment | After 1 week | After 2 weeks | After 3 weeks | After 4 weeks |  |
| G1 | 0.46 | 16.5 | 28.2±1.6 | 6.4±0.2 | 4.6±0.2 | 5.2±0.1 | 17.5±4.0 | 35.8±16.5 |
| G2 | 0.7 | 60.0 | 27.6±2.0 | 23.5±1.4 | 24.9±1.3 | 24.1±0.9 | 8.7±1.6 | 66.4±7.0 |
| G3 | 1.1 | 10.7 | 28.8±1.7 | 19.2±0.5 | 23.2±0.6 | 17.4±1.8 | 7.4±1.6 | 72.2±5.0 |
| G4 | 1.7 | 39.0 | 28.1±1.6 | 29.2±1.7 | 19.0±1.9 | 16.0±1.6 | 7.6±2.7 | 71.2±11.1 |
| G5 | 2.6 | 7.0 | 28.0±1.9 | 26.9±1.9 | 20.3±2.6 | 17.6±1.2 | 3.6±0.5 | 87.9±1.6 |
| G6 | 4 | 25.4 | 29.1±1.7 | 22.3±0.8 | 22.5±0.6 | 15.0±1.4 | 5.6±1.1 | 79.5±4.7 |

PZI, protamine zinc insulin; Se, sodium selenite; FBG, fasting blood glucose; G1-G6, group1-group6. Results are shown as means ± SEM.

**Supplementary Table 2.** Optimization formulations analysis of PZI and Se

|  | Standardized dose | b(d1) | b(d1d2) | *P* value | Optimized Dose | Annotation |
| --- | --- | --- | --- | --- | --- | --- |
| PZI | d1 | 3.846 | -- | 0.030 | 4.000 | Dmax of PZI |
| Na_2_SeO_3_ | d2 | 0.151 | -- | 0.086 | 7.000 | Dmax of Na_2_SeO_3_ |
| Mutual element | d1d2 | -- | 0.642 | 0.062 | 7.000 | Dmax of Na_2_SeO_3_  The two components are additive. |
| Degree of importance | | PZI > Na_2_SeO_3_ | | | | |
| Theoretical optimal dosage | | Dosage of PZI : 4.0 U/kg/d  Dosage of Na_2_SeO_3_ : 7.0μg/kg/d | | | | |
| Theoretical optimal proportion | | PZI : Na_2_SeO_3_ = 1.00 : 1.75 | | | | |

PZI, protamine zinc insulin; FBG, fasting blood glucose; d, standardized dose; b, the coefficient of standardized dose. The value of b(d) reflected the relative importance of each component. The value of b(d) reflects the relative importance of each component. The greater the value of b(d) is, the more significant the dose-dependent manner is. The component with maximum b(d) acts as the main drug. b(d1d2) means the interaction between the two drugs, the greater the value is, the better the synergistic effect is. Data analysis showed that the optimal proportion is 1.00: 1.75 (PZI 4 U/kg/d: Se 7μg/kg/d) by DAS 1.0 software.
